# Lipids, lipid-modified drug target genes, and the risk of male infertility: a Mendelian randomization study

**DOI:** 10.3389/fendo.2024.1392533

**Published:** 2024-07-24

**Authors:** Wei Li, Hu Li, Cheng Zha, Bangwei Che, Ying Yu, Jianjun Yang, Tao Li

**Affiliations:** ^1^ Department of Urology, the Affiliated Hospital of Guizhou Medical University, Guiyang, China; ^2^ Emergency Department, Affiliated Hospital of Binzhou Medical College, Binzhou, China; ^3^ Department of Urology, The First Affiliated Hospital of Guizhou University of Traditional Chinese Medicine, Guiyang, China

**Keywords:** male factor infertility, lipids, drug target mendelian randomization, lipid-lowering drugs, vitamin D

## Abstract

**Background:**

Previous observational studies have reported a possible association between circulating lipids and lipid-lowering drugs and male infertility (MIF), as well as the mediating role of circulating vitamin D. Then, due to issues such as bias, reverse causality, and residual confounding, inferring causal relationships from these studies may be challenging. Therefore, this study aims to explore the effects of circulating lipids and lipid-lowering drugs on MIF through Mendelian randomization (MR) analysis and evaluate the mediating role of vitamin D.

**Method:**

Genetic variations related to lipid traits and the lipid-lowering effect of lipid modification targets are extracted from the Global Alliance for Lipid Genetics Genome-Wide Association Study. The summary statistics for MIF are from the FinnGen 9th edition. Using quantitative expression feature loci data from relevant organizations to obtain genetic variations related to gene expression level, further to explore the relationship between these target gene expression levels and MIF risk. Two-step MR analysis is used to explore the mediating role of vitamin D. Multiple sensitivity analysis methods (co-localization analysis, Egger intercept test, Cochrane’s Q test, pleiotropy residuals and outliers (MR-PRESSO), and the leave-one-out method) are used to demonstrate the reliability of our results.

**Result:**

In our study, we observed that lipid modification of four lipid-lowering drug targets was associated with MIF risk, the LDLR activator (equivalent to a 1-SD decrease in LDL-C) (OR=1.94, 95% CI 1.14-3.28, FDR=0.040), LPL activator (equivalent to a 1-SD decrease in TG) (OR=1.86, 95% CI 1.25-2.76, FDR=0.022), and CETP inhibitor (equivalent to a 1-SD increase in HDL-C) (OR=1.28, 95% CI 1.07-1.53, FDR=0.035) were associated with a higher risk of MIF. The HMGCR inhibitor (equivalent to a 1-SD decrease in LDL-C) was associated with a lower risk of MIF (OR=0.38, 95% CI 0.17-0.83, FDR=0.39). Lipid-modifying effects of three targets were partially mediated by serum vitamin D levels. Mediation was 0.035 (LDLR activator), 0.012 (LPL activator), and 0.030 (CETP inhibitor), with mediation ratios of 5.34% (LDLR activator), 1.94% (LPL activator), and 12.2% (CETP inhibitor), respectively. In addition, there was no evidence that lipid properties and lipid modification effects of six other lipid-lowering drug targets were associated with MIF risk. Multiple sensitivity analysis methods revealed insignificant evidence of bias arising from pleiotropy or genetic confounding.

**Conclusion:**

This study did not support lipid traits (LDL-C, HDL-C, TG, Apo-A1, and Apo-B) as pathogenic risk factors for MIF. It emphasized that LPL, LDLR, CETP, and HMGCR were promising drug targets for improving male fertility.

## Introduction

Male infertility (MIF) refers the failure to achieve pregnancy after 12 months of regular unprotected sexual intercourse due to male factors. It troubles 10–20% of couples worldwide and contribute tremendous obstacles to social and economic progress (1, 2). Meanwhile, as the concentration and quality of male sperm continue to decline, more and more couples will be troubled (1, 3). There are many reasons for MIF, including congenital, acquired, idiopathic, or environmental factors, such as fragmented sperm DNA, which can not only affect the quality of sperm and lead to infertility but also promote recurrent miscarriage or recurrent implantation failure in female partners, according to the latest research (4–6). However, despite extensive research, little is known about the exact mechanism of MIF (1, 3), which poses a great challenge for developing effective treatment methods.

Circulating lipids are a collective term for apolipoprotein A (APOA), apolipoprotein B (APOB), high-density lipoprotein cholesterol (HDL-C), low-density lipoprotein cholesterol (LDL-C), and triglycerides in the blood, which have complex and unclear relationship with male fertility. Cholesterol, as the main substrate for steroid synthesis, has shown a crucial role in steroid hormone generation and relates to downstream effects (including spermatogenesis, empowerment, and penetration of the zona pellucida to fertilize oocytes) (7–9). Its content in the sperm membrane is directly related to normal sperm morphology and fertility, indicating that circulating lipids might have protective effects on MIF. This has been confirmed in some observational studies (10, 11) but more studies reported opposite conclusions (12–14), and the causal relationship between circulating lipids and the risk of MIF has not been fully determined.

Statins are the most commonly used lipid-lowering drugs and are widely used in cardiovascular diseases. It has been reported that statins can improve fertility by correcting lipid metabolism disorders through various molecular pathways; however, other studies revealed that statins led to MIF by impairing semen quality and decreasing gonadal function (15–17); in addition, some authors reported statins have no impact on male fertility (18, 19). Thus, the causal relationship between lipid-lowering drugs and MIF risk remains uncertain. It is well known that the treatment of male infertility depends on the development of new drugs, and the important path of new drug development is the reuse of drugs, as this can reduce the cost of new drug research and greatly accelerate the usually lengthy approval process. In addition, as the increasing use of lipid-lowering drugs and new lipid-lowering drugs, it is necessary to elucidate the specific causal relationship between lipid-lowering drugs and MIF risks.

Vitamin D is an endogenous hormone related to human health (20, 21). Previous studies have shown that vitamin D has a significant protective effect on MIF. Interestingly, statins can affect serum vitamin D levels and are considered one of the causes of adverse reactions to statins (22, 23), and vitamin D supplements have been shown to have a synergistic effect with statins, effectively alleviating adverse reactions (24, 25). Indicating that serum vitamin D levels may play a crucial role in the impact of lipid-lowering drugs on MIF. However, there is currently a lack of relevant research.

In recent years, Mendelian randomization (MR) analysis has become a popular and effective causal reasoning method. It uses genetic variations (single nucleotide polymorphisms, SNPs) as instrumental variables (IV) to infer causal relationships between outcomes and exposure, effectively avoiding confounding biases in traditional epidemiological studies and providing a valuable alternative to randomized clinical trials (26). MR analysis of drug targets is a method of simulating genetic variations in pharmacological inhibition (enhancement) of drug-gene targets. The regression estimates obtained from this analysis reflect the long-term effects of drug use, can be used to infer the causal relationship between drug use and disease (27).

## Method

### Research design

**Figure 1:**
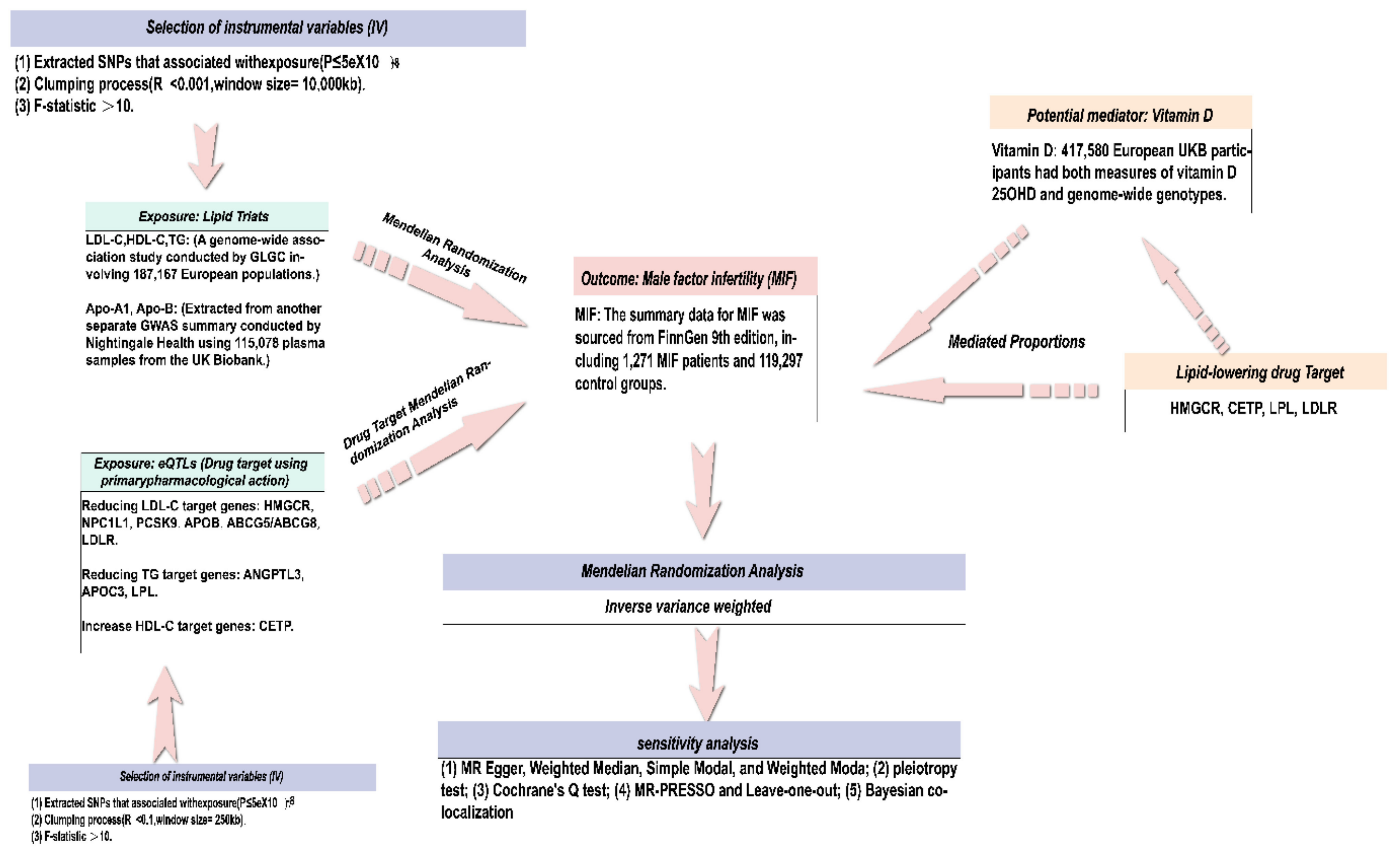
Overview of the research design. The figure was created with Adobe Illustrator.

The design of this study referred to the Mendelian Randomized Enhanced Epidemiological Observation Study Report (STROBE-MR) (28). The study design is visually represented in **Figure 1**, offering a comprehensive depiction. To obtain the necessary data, publicly accessible summary-level data from genome-wide association studies (GWAS) and expression quantitative trait loci (eQTL) studies were used. In addition, FDR (False Discovery Rate) correction is used to eliminate the increase in Class I errors caused by multiple test corrections. All data used in this work is from studies with subject consent and ethical recognition; therefore, our study does not require ethical approval from the institutional review committee. All analyses in this study, including Mendelian randomization (MR) and Bayesian co-localization, were conducted using R. The “TwoSampleMR” package is used for MR analysis, while the “Coloc” package is used for Bayesian co-localization analysis.

### Data sources

In this study, the summarized GWAS data for MIF was sourced from FinnGen 9th edition, including 1,271 MIF patients and 119,297 control groups, all participants with European ancestry. Cases of MIF are identified using diagnostic codes from the International Classification of Diseases codes 8, 9, and 10 (including azoospermia, oligospermia, extratesticular infertility, and unspecified male infertility). The summarized GWAS data on lipid traits (low-density lipoprotein, high-density lipoprotein, and cholesterol) was obtained from a genome-wide association study conducted by the Global Lipids Genetics Consortium (GLGC) involving 187,167 European populations (29). The summarized GWAS data for Apolipoprotein A1 and Apolipoprotein B was extracted from another separate GWAS summary conducted by Nightingale Health using 115,078 plasma samples from the UK Biobank (30).

### Genetic instrument selection

In order to obtain genetic tools that meet the three major hypotheses of MR analysis, we adopted a series of strict standards (26): (1) Select SNPs with genome-wide significance (p<5 × 10-8), with an acceptable mutation probability (secondary allele frequency>1%); (2) Execute clump (r ^ 2<0.001, kb=10000 kb) to eliminate linkage imbalance between genetic instruments, (3) remove palindrome genetic instruments when palindrome genetic instruments exist; (4) The F-statistic is used to estimate the strength of each genetic instruments and select all strong instrumental variables (F>10). the formula is R^ 2×(N − 2)/(1 − R^ 2), where R^ 2 is the cumulative explained variance of selected SNPs in exposure that used (2×EAF×(1 − EAF)×beta^ 2)/[(2×EAF×(1 − EAF) ×beta^ 2) + (2×EAF×(1 − EAF)×N×SE(beta) ^ 2)], where N is the sample size of research, EAF is the effect allele frequency, beta is the estimated genetic effect, and SE(beta) is the standard error of the beta.

### Genetic instrumental variables for lipid modifying targets

According to the guidelines for managing dyslipidemia, selecting common lipid-lowering drugs and novel therapies, and further obtaining closely related pharmacological targets in the DrugBank database (https://go.drugbank.com/), these target genes were divided into target genes that reduce LDL-C (HMGCR, NPC1L1, PCSK9, APOB, ABCG5/ABCG8, LDLR), increase HDL-C (CETP), and decrease TG (ANGPTL3, APOC3, LPL) based on their main pharmacological effects. Furthermore, to simulate the lipid modification effect of these targets, we followed the methods used in previous studies (31, 32), utilized summary-level data from GWAS of lipid traits (LDL-C, HDL-C, and TG) to determine the genetic instrument variables of these drugs. We selected SNPs within the 250kb physical window region of these genes at the genome-wide significance level. Afterward, we performed a clump (r ^ 2<0.2, kb=250 kb) to eliminate linkage disequilibrium between them. In addition, for drug targets associated with MIF risk, further use of publicly expressed quantitative trait loci (eQTL) data from eQTLgen to identify cis eQTLs significantly associated with drug target gene expression levels These cis eQTLs must meet strict criteria, including significance thresholds P<5 * 10-8 and r2<0.1.

### MR analysis

In this study, the random effects model inverse variance weighting (Re-IVW) was used as the main analysis method, which can combine the causal effects of individual genetic instruments, allowing for heterogeneity between genetic instruments, and returning unbiased estimates of causal relationships when all genetic instruments were valid and pleiotropy levels were balanced. It is considered the simplest and most reliable method in MR analysis. To enhance the robustness of MR analysis, we use four additional analysis methods (MR Egger, Weighted Median, Simple Modal, and Weighted Modal) to supplement the results. Subsequently, various sensitivity analyses were used to confirm the reliability of our results, including the Egger intercept test for assessing the presence of horizontal pleiotropy, the Cochrane’s Q test for assessing heterogeneity among included SNPs, and the pleiotropy residuals and outliers (MR-PRESSO) and Leave-one-out method for identifying and excluding genetic instruments with potential pleiotropy (p<0.05). In addition, in order to address the possibility that variations closely related to real causal variations may affect results through non-lipid pathways, we conducted Bayesian co-localization analysis, which helps to detect the possibility of genetic confounding by evaluating the posterior probabilities of different causal variations, shared causal variations, and co-localization probabilities. This analysis provides several outputs of interest. This includes the probability of independent causal variation between exposure and outcome features (H3), as well as the probability of shared causal variation affecting both features (H4). The main output we are interested in is the probability of co-localization, which is calculated using the formula H4/(H3+H4), which indicates the degree to which the same genetic variation affects MR analysis results.

### Intermediate MR analysis

We use a two-step MR method for intermediate MR analysis (33, 34). In the first step, we calculated the causal effect of lipid-modifying effect of targets on the mediator (vitamin D). In the second step, we estimated the causal effect of the mediator (vitamin D) on MIF (β 2). By formula (β 1* β 2) Calculate the mediating effect of vitamin D, subtract the mediating effect of vitamin D from the causal effect of lipid-modifying effect of targets on MIF, and obtain the direct effect of lipid-modifying effect of targets on MIF. In addition, dividing the mediating effect by the causal effect of lipid-modifying effect of targets on MIF yields the proportion mediated by vitamin D.

## Result

### Lipid traits and MIF

Instrumental variables for lipid traits including HDL-C, LDL-C, TG, Apo-A1, and Apo-B were presented in the supplementary table. The F-statistics of each genetic instrument were all greater than 10, indicating that instrument bias is unlikely to affect the reliability of the analysis results (**Supplementary Tables S4-8**).

**Figure 2:**
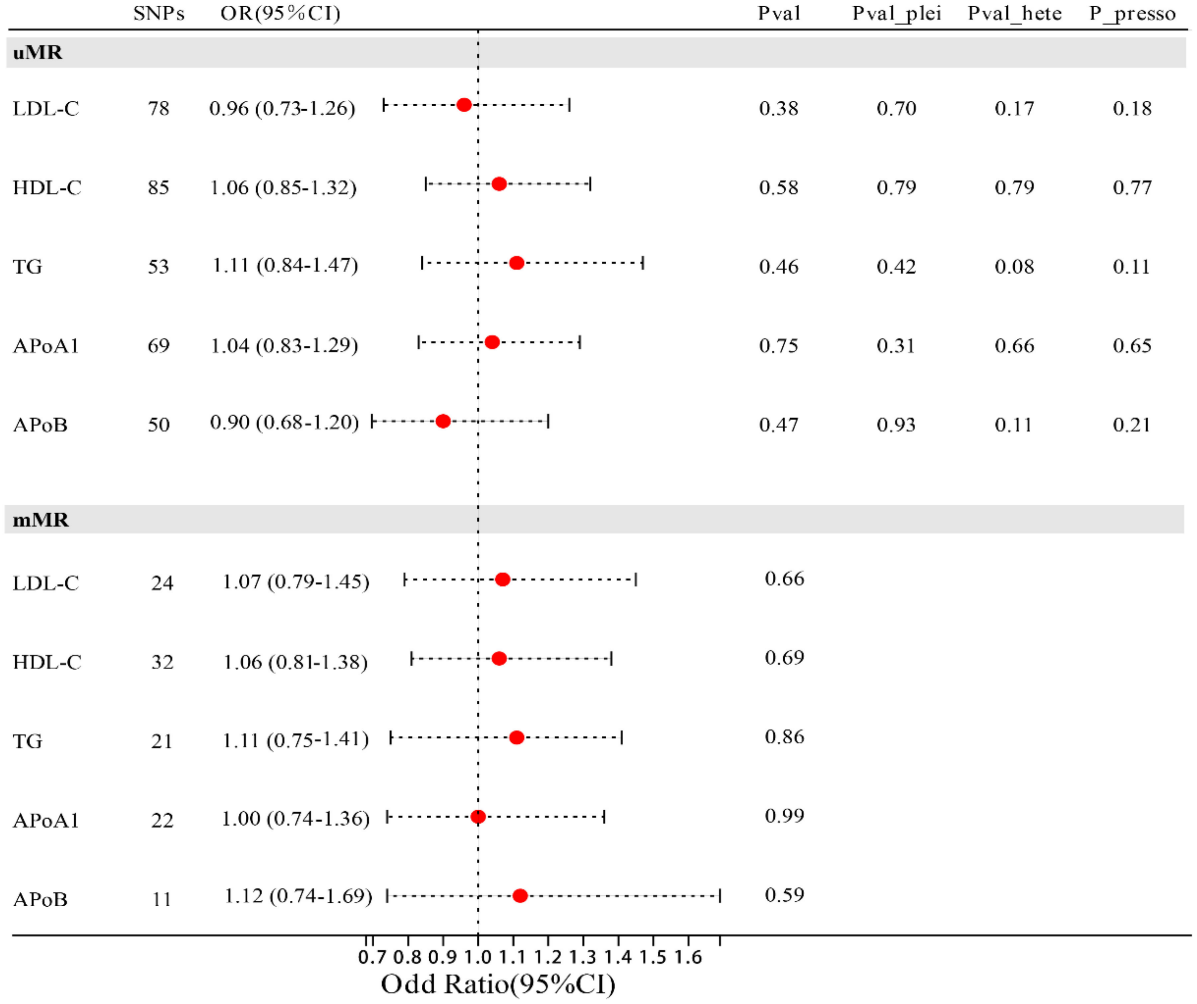
Forest plot of association of lipid traits with risk of MIF. uMR, univariate MR analysis; mMR, multi-factor MR analysis; Pval_plei is the result of Egger intercept test; Pval_hete is the result of Cochrane’s Q test; Pval_presso is the result of MR-PRRESSO.

In univariate MR analysis, we did not observe a causal relationship between lipid traits and MIF (**Figure 2**), with LDL-C (OR=0.92; 95% CI: 0.76-1.11; p=0.378), HDL-C (OR=1.06; 95% CI: 0.85-1.32; p=0.581), TG (OR=1.11; 95% CI: 0.84-1.47; p=0.458), Apo-B (OR=0.90; 95% CI: 0.68-1.20; p=0.474), and Apo-A1 (OR=1.04; 95% CI: 0.83-1.29; p=0.754). After adjusting these risk factors (including diabetes, coronary heart disease, and body mass index) related to MIF, multivariable MR analysis still did not observe the causal relationship between lipid traits and MIF with genetic prediction, in which LDL-C (OR=1.07; 95% CI: 0.79-1.45; p=0.664), HDL-C (OR=1.06; 95% CI 0.81-1.38; p=0.691), TG (OR=1.03; 95% CI: 0.75-1.41; p=0.691), Apo-B (OR=1.12; 95% CI: 0.74-1.69; p=0.586), Apo-A1 (OR=1.00; 95% CI: 0.74-1.36; p=0.990). Sensitivity analysis supports the reliability of the research results (**Figure 2**). The Egger intercept test method did not detect the presence of horizontal pleiotropy (all p values for intercept > 0.05). Similarly, Cochrane’s Q test did not detect heterogeneity (all p > 0.05), and the Leave-one-out method and MR-PRRESSO method did not find the presence of genetic instruments that affect the reliability of the results.

### Genetically predicted lipid-modifying effect of targets and MIF risk

**Supplementary Table S2** lists the instrumental variables for lipid-modifying effect of ten targets, including HMGCR inhibitor, NPC1L1 inhibitor, PCSK9 inhibitor, APOB blocker, ABCG5 and ABCG8 activator, LDLR activator, ANGPTL3 inhibitor, APOC3 blocker, CETP inhibitor, and LPL activator. Similarly, the F-statistics of all genetic instruments exceed the threshold of 10, indicating that instrument bias is unlikely to affect the reliability of our analysis results (**Supplementary Table S3**).

**Figure 3:**
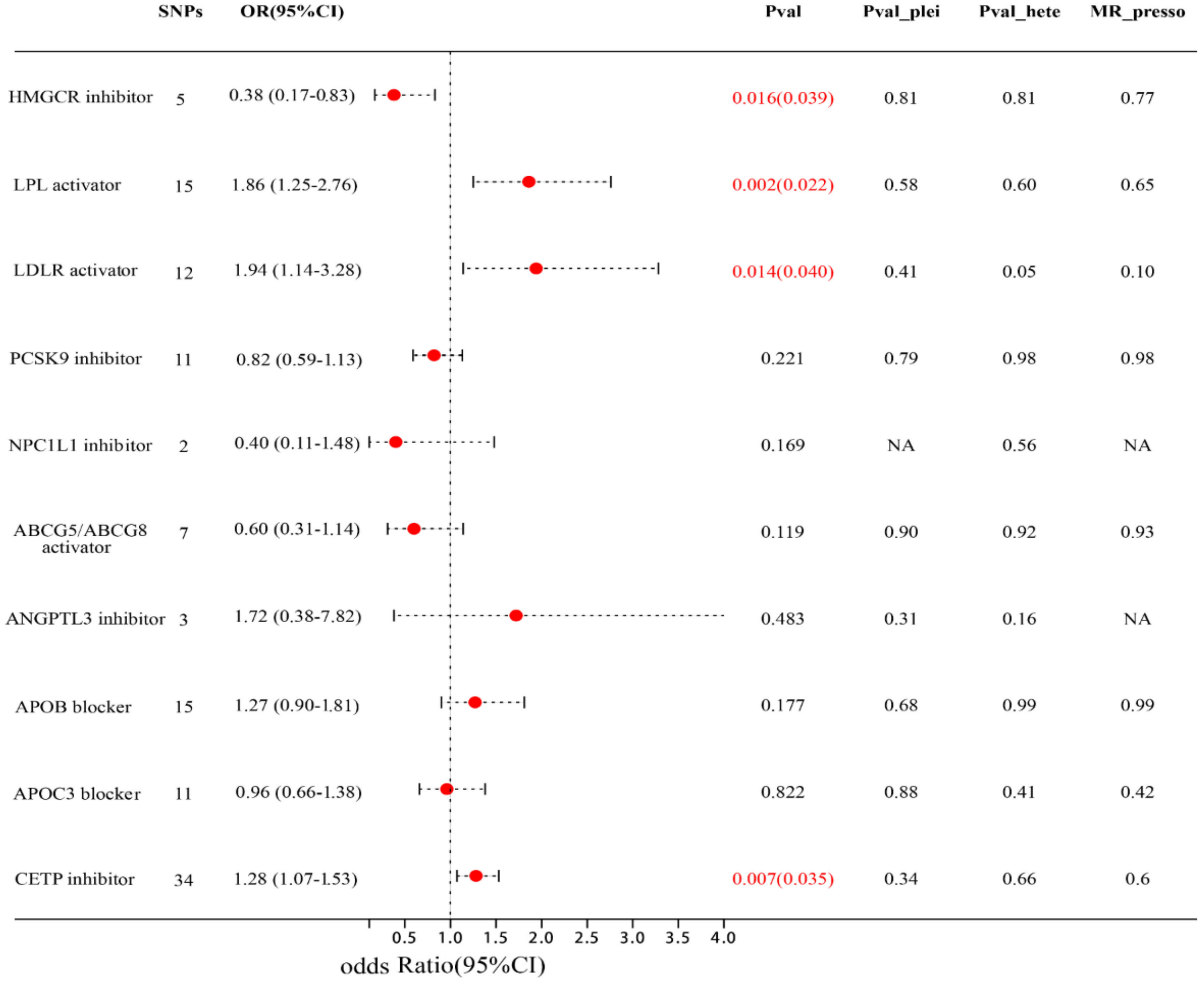
Forest plot of association of lipid-modifying effect of targets and MIF risk. Pval_plei is the result of Egger intercept test; Pval_hete is the result of Cochrane’s Q test.

**Figure 3**; **Supplementary Table S10** showed the associations between 10 lipid-lowering drug classes and MIF risk, LDLR activator (equivalent to a 1-SD decrease in LDL-C), LPL activator (equivalent to a 1-SD decrease in TG), and CETP inhibitor (equivalent to a 1-SD increase in HDL-C) were associated with a higher risk of MIF, with LDLR activator: (OR=1.94; 95% CI: 1.14-3.28; FDR=0.039), LPL activator: (OR=1.86; 95% CI: 1.25-2.76; FDR=0.022), CETP inhibitor: (OR=1.28; 95% CI: 1.07-1.53; FDR= 0.035). HMGCR inhibitor (equivalent to a 1-SD decrease in LDL-C) is associated with a lower risk of MIF (OR=0.38; 95% CI: 0.17-0.83; FDR=0.039). The other, lipid-modifying effects of six targets, including NPC1L1 inhibitor, PCSK9 inhibitor, APOB blocker, ABCG5 and ABCG8 activator, ANGPTL3 inhibitor, and APOC3 blocker, are not correlated with the risk of MIF. Subsequently, we did not observe any heterogeneity among them (all p > 0.05), and the Egger intercept test method did not detect the presence of horizontal pleiotropy (all p values for intercept > 0.05). Furthermore, the leave-one-out and MR-PRRESSO methods did not detect any abnormal instrumental variables with potential pleiotropy.

### The expression of lipid-modified targets gene and MIF

Here, we analyzed the expression levels of the four lipid modification targets mentioned above. We obtained cis-eQTLs significantly correlated with drug target gene expression levels from publicly available eQTLs (whole blood tissue) and further analyzed three drug targets (HMGCR, CETP, and LPL). LDLR is excluded in subsequent analysis due to the lack of available cis-eQTLs. Our results showed that HMGCR expression levels were associated with lower MIF risk (OR = 0.56; 95% CI 0.36-0.87; FDR = 0.030), while gene expression levels of LPL and CETP were not associated with MIF risk.

### Colocalization

To further validate the results’ reliability, additional co-localization analysis was used to verify whether our analysis was affected by confounding LD variations. Our results showed that for the LDL-C and MIF risks within the HMGCR gene, the probability of individual causal variations (0.05%) was much lower than the probability of shared causal variations (13.5%), while the probability of co-localization was 96.1%; The posterior probabilities of TG and MIF risks within the LPL gene are 2.30%, 7.75%, and 77.1%, respectively; The posterior probabilities of HDL-C and MIF risk within the CETP gene are 0.61%, 2.43%, and 79.9%, respectively; The posterior probabilities of LDL-C and MIF risk within the LDLR gene are 2.23%, 5.09%, and 69.5%, respectively. These results indicate that our results are unlikely to be affected by confounding LD variations (**Table 1**).

**Table 1:** Colocalisation results.

Drug targets	H0	H1	H2	H3	H4	H4/(H3+H4)
CETP	0.00E+00	9.70E-01	0.00E+00	6.14E-03	2.43E-02	7.99E-01
HMGCR	5.10E-79	8.59E-01	3.31E-81	5.44E-03	1.35E-01	9.61E-01
LDLR	9.88E-280	9.27E-01	2.38E-281	2.23E-02	5.09E-02	6.95E-01
LPL	2.10E-197	9.00E-01	5.39E-199	2.30E-02	7.75E-02	7.71E-01

Posterior probability for H0: neither trait has a genetic association in the region; H1: only trait 1 has a genetic association in the region; H2: only trait 2 has a genetic association in the region; H3: both traits are associated, but with different causal variants; H4: both traits are associated and share a single causal variant. H4/(H3+H4) represents the probability of colocalization conditional on the presence of a causal variant for the outcome.

### Exploring the mediation of serum vitamin D levels by two-step MR analysis

We attempted to use a two-step MR analysis to explore the mediation of vitamin D in the lipid-modifying effect of four targets (HMGCR inhibitor, LPL activator, LDLR activator, CETP inhibitor) and MIF. In the first step of MR analysis, we found that LDLR activator, LPL activator, and CETP inhibitor are associated with a decrease in serum vitamin D levels, with LDLR activator:(β = - 0.091; 95% CI: -0.106 to -0.076; p=3.75e-10), LPL activator:(β = - 0.031; 95% CI: -0.044 to -0.020; p=0.009), CETP inhibitor:(β = - 0.076; 95% CI: -0.081 to -0.071; p=1.60e-45). Subsequently, the results of the second MR analysis were consistent with previous reports, indicating that serum vitamin D levels were associated with a lower risk of MIF (OR=0.68; 95% CI: 0.52-0.88; p=0.003). Further analysis indicates that three lipid-modifying effects of targets are partially mediated by serum vitamin D levels. Mediation is 0.035 (LDLR activator), 0.012 (LPL activator), and 0.030 (CETP inhibitor), with mediation ratios of 5.34% (LDLR activator), 1.94% (LPL activator), and 12.2% (CETP inhibitor), respectively (**Table 2**).

**Table 2:** The mediating role of vitamin D.

Drug targets	Total effect	Indirect (mediating) effect	Direct effects	Mediation effect ratio (%)
CETP	0.25	0.03	0.22	12.2
LDLR	0.66	0.035	0.635	5.34
LPL	0.62	0.012	0.608	1.93

## Discussion

This study provides convincing evidence supporting the association of LDLR activator, LPL activator, and CETP inhibitor with higher MIF risk, while HMGCR inhibitor with lower MIF risk; suggesting that LDLR, LPL, CETP, and HMGCR may be potential targets for preventing MIF. Moreover, the increased or reduced risk seemed not to LDL-C or TG control, as no clear evidence suggested a general impact of lipid traits on MIF risk. In addition, the study confirmed the robustness of the results through detailed heterogeneity and sensitivity analysis methods. Intermediary analysis indicated the mechanisms of LDLR activator, LPL activator, and CETP inhibitor on MIF partially by regulating serum vitamin D levels.

Cholesterol is the main source of sex steroid hormone synthesis and plays a decisive role in steroid production and spermatogenesis (8, 9). Animal studies have shown a correlation between cholesterol or steroid production and male fertility (35), however, these animal-model-based evidence do not seem to apply to humans considering complex relationship between lipids and sperm production. In our study, there is no evidence supporting the association between lipid characteristics (LDL-C, HDL-C, TG, Apo-A1, and Apo-B) and MIF risk, this is consistent with views of Neergaard (36) and Osadchuk (37) whom believes that lipid levels in seminal plasma are positively correlated with sperm quality rather than lipid levels. Lipids in seminal plasma may not originate from the blood but from the epithelial cells of male reproductive tract (36). In addition, enzymes involved in cholesterol metabolism in human reproductive tract, testicular interstitial cells, supporting cells, and mature germ cells can self-regulate local cholesterol levels (36). Therefore, lipid characteristics are unlikely to affect sperm quality. However, some studies reported opposite conclusions (10–14) that blood lipid levels were correlated to sperm quality, although some shortcomings may lead to false positive results. Firstly, participants selection is likely biased rather than random, especially in cross-sectional designs. Secondly, these populations seek medical attention due to fertility-related issues, which affects the reliability of the results. Finally, the lack of important information further affects the reliability of the results, such as exercise habits, dietary patterns, and family history.

The relationship between lipid-lowering drugs and MIF mainly focuses on statins. Statins can effectively reduce the level of LDL-C by inhibiting activity of HMG-CoA reductase (38). Our analysis confirms for the first time that HMGCR inhibitors and HMGCR gene expression levels are associated with lower MIF risk. This seems to be unrelated to the lipid-lowering ability of HMGCR inhibitors, as we did not observe any association between lipid characteristics and male infertility, indicating that other factors mediate this positive effect. HMGCR inhibitors have shown ability to reduce production of inflammatory mediators and cytokines to alleviate inflammatory responses (39, 40). These effects may relate to the protective effect of HMGCR inhibitors, as it found that male infertility patients typically had elevated levels of certain inflammatory markers, such as C-reactive protein, interleukin-6, and tumor necrosis factor-α. This might indicate a potential systemic inflammatory state (37, 38). In addition, previous studies have shown that statins can inhibit oxidant-induced reactive oxygen species (ROS) (41, 42). The increased ROS can lead sperm dysfunction as well as abnormalities in sperm quantity, motility, and morphology (43). Meanwhile, the imperfect antioxidant mechanism of sperm makes them particularly sensitive to damages induced by excessive ROS (44). However, previous observational studies have almost reported the negative effects of statins (16–19). Although Yan (45) confirmed a correlation between HMGCR inhibitors and lower serum testosterone levels (including total testosterone and bioavailable testosterone), this did not mean that it could lead abnormal semen quality or even MIF. There is no clear relationship between testosterone and function, nor between serum testosterone levels and semen parameters (46, 47). On the contrary, the abuse of exogenous androgens can lead to MIF (48). Therefore, we speculated that this negative impact may be related to reverse causality and residual confounding effects, as well as upregulation of LDLR and LPL expression or activity.

LDLR, as an indirect target of statins, increases in activity and expression with HMGCR inhibition (49). We also firstly confirmed that LDLR activators were associated with higher risk of MIF. LDLR is a key cell surface receptor for cholesterol uptake and transport, which regulating the homeostasis of plasma and cellular cholesterol by mediating the up-taking and metabolism of plasma-derived LDL-C (49). It is related to the process of sperm production (36, 50, 51). Research has found that heterozygous deletion of LDLR reduced cholesterol toxicity in the testes of hamsters and improved the reproductive ability of male hamsters, indicating the presence of sufficient cholesterol and increased LDR level led the accumulation of cholesterol in the testes, which finally inducing testicular toxicity. This suggests that LDLR may regulate cholesterol metabolism and stability in the reproductive system (50). This has also been confirmed in the study by Pelletier et al. (51), who found that when the upstream inhibitor of LDLR (PCSK9) was missing, the increased LDLR contributed excessive accumulation of testicular cholesterol to affect metabolic stability and immune tolerance in the seminiferous tubules, and finally impairing mice fertility. Similarly, statin drugs have been reported to increase the expression level and activity of LPL (52), our study also showed that LPL activators were associated with higher MIF risk, indicating that LPL might be involved in the negative effects of statins. However, no research has focused on the relationship between LPL and MIF. We speculated that it was related to the stable imbalance of fatty acid metabolism in reproductive tract tissues. Firstly, LPL is a key enzyme in lipid metabolism. As a gatekeeper of tissue fatty acid uptake (53), it is widely expressed in male reproductive tissues (36). Upregulation of LPL activity and expression will lead increased breakdown of triglycerides, production of more free fatty acids, and entry into reproductive tract tissues, leading to the accumulation of free fatty acids and activation of inflammatory pathways (53), which will further affect sperm quality (54). However, other studies involving *in vitro* experiments, animal models, and clinical trials are needed to reveal complex molecular pathways and confirm the therapeutic potential of LPL regulation in reducing MIF.

Vitamin D is an endogenous hormone that associates to human health (20, 21). Previous studies have shown that statin drugs can affect serum vitamin D level and are considered one of the causes of adverse reactions to statins (22, 23). On the contrary, vitamin D supplements have been shown to have a synergistic effect with statins to effectively alleviate adverse reactions (24, 25). Therefore, the effect of statins on serum vitamin D levels may also be involved in the negative impact on MIF. In our analysis, we found no correlation between HMGCR inhibition and vitamin D levels. LDLR activators and LPL activators were associated with lower serum vitamin D levels, which may be the reason why statins were associated with lower vitamin D levels. However, the increased serum vitamin D level is considered a protective factor for MIF (22). Therefore, we believe that the decreased serum vitamin D may also be involved in the negative effects of statins on MIF.

CETP mediates the bidirectional transfer of cholesterol esters and triglycerides between plasma lipoprotein particles, which receives great attention due to its activity to increase HDL-C (55). However, CETP inhibitors have always been a controversial topic that previous clinical trials reported it increased risk of major coronary heart disease events (56) due to the renin-angiotensin-aldosterone system (RASS) (57), meanwhile, as overactivated RASS system is also associated with MIF (58), some studies suggested that the CETP inhibitors were theoretically increased MIF risk, which is consistent with our current finding. Recently, more attention has been paid on developing new drugs with CETP inhibitors as the core mechanism, indicating exploring the specific mechanisms of CETP and MIF will be of great significance.

Our research provides several advantages. Firstly, this is the first study to explore the causal relationship between lipid traits, lipid modification targets, and MIF. Secondly, we used univariate MR analysis, multivariate MR analysis, and drug target MR analysis to investigate the causal relationship between LDLR activator, LPL activator, CETP inhibitor, HMGCR inhibitor, and MIF, as well as the mediation of serum vitamin D levels to emphasize the necessity of further studying the potential reproductive effects of long-term use of lipid-lowering drugs. Meanwhile, our research also suggests that further research is needed to investigate the relationship between other novel drug targets with synergistic effects with lipid-lowering drugs and MIF, such as TNF - α (59, 60), which possibly further the discovery of novel therapeutic targets for MIF. In addition, the MR design is not easily affected by residual clutter, and the GWAS summary level data in this study has a large sample size, which effectively reduces the confounding factors. Finally, the detailed heterogeneity and multi-validity tests were conducted to confirm the reliability of our results.

However, several limitations also exist. For example, as genetic variations typically originate from parents and are less susceptible to environmental influences, they reflect a lifelong impact on the exposure. Therefore, using genetic variations to study the effects of lipid-lowering drugs is limited, reflecting the long-term effects of lipid-lowering drugs on MIF, which may not be comparable to the short-term effects of lipid-lowering drugs (within a specific time frame). So, the drug MR analysis helps to determine the direction of causal relationships rather than quantifying the degree of correlation. So, RCT research under specific conditions is still necessary. Meanwhile, despite with detailed heterogeneity and pleiotropy tests, the effect of horizontal pleiotropy cannot be completely ruled out. In addition, due to the significant differences in genetic variation among different races and the fact that our research findings are mainly related to individuals of European ancestry, these findings may not necessarily apply to other ethnic groups. Therefore, it is necessary to conduct cross racial analysis when appropriate GWAS data is available. Finally, despite the large sample size, the original GWAS data was not stratified according to certain subtypes (MIF caused by sperm quality, MIF caused by testicular disease, and obstructive MIF, etc.), therefore, this study was unable to conduct stratified analysis. It remains a research topic and should be considered when specific datasets are available in the future.

## Conclusion

This study does not support lipid traits (LDL-C, HDL-C, TG, Apo-A1, and Apo-B) as pathogenic risk factors for MIF. However, it emphasizes that LPL, LDLR, CETP, and HMGCR are promising drug targets for improving male fertility. The underlying mechanisms should be elucidated in further research, and the role of LPL activators, LDRL activators, CETP inhibitors, and HMGCR inhibitors in MIF in basic or even clinical trials might be worth assessing. However, the findings in this study require a larger GWAS dataset with a more complete phenotype, as well as other potentially relevant genetic variations, to validate.

## Data availability statement

The original contributions presented in the study are included in the article/**Supplementary Material**. Further inquiries can be directed to the corresponding authors.

## Author contributions

WL: Conceptualization, Data curation, Methodology, Supervision, Writing – original draft, Writing – review & editing. BC: Conceptualization, Investigation, Methodology, Writing – original draft. HL: Data curation, Formal analysis, Validation, Visualization, Writing – original draft, Writing – review & editing. CZ: Investigation, Writing – original draft. YY: Conceptualization, Writing – review & editing. JY: Investigation, Project administration, Writing – original draft. TL: Conceptualization, Data curation, Funding acquisition, Investigation, Methodology, Resources, Supervision, Validation, Visualization, Writing – original draft, Writing – review & editing.
